# Low circulating microRNA levels in heart failure patients are associated with atherosclerotic disease and cardiovascular-related rehospitalizations

**DOI:** 10.1007/s00392-017-1096-z

**Published:** 2017-03-14

**Authors:** Eline L. Vegter, Ekaterina S. Ovchinnikova, Dirk J. van Veldhuisen, Tiny Jaarsma, Eugene Berezikov, Peter van der Meer, Adriaan A. Voors

**Affiliations:** 1 Department of Cardiology, AB31, University Medical Center Groningen, Hanzeplein 1, 9713 GZ Groningen, The Netherlands; 2 European Research Institute for the Biology of Ageing, University of Groningen, Antonius Deusinglaan 1, 9713 AV Groningen, The Netherlands; 3 Faculty of Medical and Health Sciences, University of Linkoping, 581 83 Linköping, Sweden

**Keywords:** Circulating microRNAs, Heart failure, Atherosclerosis, Biomarkers, Rehospitalization

## Abstract

**Objective:**

Circulating microRNAs (miRNAs) have been implicated in both heart failure and atherosclerotic disease. The aim of this study was to examine associations between heart failure specific circulating miRNAs, atherosclerotic disease and cardiovascular-related outcome in patients with heart failure.

**Methods:**

The levels of 11 heart failure-specific circulating miRNAs were compared in plasma of 114 heart failure patients with and without different manifestations of atherosclerotic disease. We then studied these miRNAs in relation to biomarkers associated to atherosclerosis and to cardiovascular-related rehospitalizations during 18 months of follow-up.

**Results:**

At least one manifestation of atherosclerotic disease was found in 70 (61%) of the heart failure patients. A consistent trend was found between an increasing number of manifestations of atherosclerosis (peripheral arterial disease in specific), and lower levels of miR-18a-5p, miR-27a-3p, miR-199a-3p, miR-223-3p and miR-652-3p (all *P* < 0.05). Target prediction and network analyses identified several interactions between miRNA targets and biomarkers related to inflammation, angiogenesis and endothelial dysfunction. Lower miRNA levels were associated with higher levels of these atherosclerosis-related biomarkers. In addition, lower miRNA levels were significantly associated with rehospitalizations due to cardiovascular causes within 18 months, with let-7i-5p as strongest predictor [HR 2.06 (95% CI 1.29–3.28), C-index 0.70, *P* = 0.002].

**Conclusions:**

A consistent pattern of lower levels of circulating miRNAs was found in heart failure patients with atherosclerotic disease, in particular peripheral arterial disease. In addition, lower levels of miRNAs were associated with higher levels of biomarkers involved in atherosclerosis and an increased risk of a cardiovascular-related rehospitalization.

**Electronic supplementary material:**

The online version of this article (doi:10.1007/s00392-017-1096-z) contains supplementary material, which is available to authorized users.

## Introduction

MicroRNAs (miRNAs) can function as important regulators of a wide range of biological processes and contribute to the development of various diseases, including heart failure [1]. These small, non-coding RNAs are potent regulators of gene expression, which function via binding to the target messenger RNA (mRNA). This in turn leads to either degradation of the mRNA or to repression of translation, resulting in a disturbed protein synthesis [2]. Extracellular miRNAs can be detected in circulating blood, and have shown to function as potential biological markers in heart failure [1, 3, 4]. A variety of studies identified differentially expressed circulating miRNAs in heart failure [5–7]. We recently reported about a panel of circulating miRNAs that consistently showed lower plasma levels in (acute) heart failure patients compared to healthy controls [7]. A gradual increase in miRNA levels was seen towards more stabilized heart failure patients, chronic heart failure patients and healthy controls.

Remarkably, no cardiac specific or cardiac-enriched miRNAs (such as miR-1, miR-133, miR-499 and miR-208) were present in this set of heart failure-related miRNAs, suggesting that the most differentially expressed miRNAs in the circulation of heart failure patients do not originate from the heart. Several studies showed that blood and endothelial cells are the major source of abundant miRNAs in the circulation [8, 9] and literature on our previously found miRNA signature in heart failure revealed potential involvement in vascular-related processes including angiogenesis, endothelial dysfunction and inflammation [10–15]. Disturbances in these processes are frequently present in patients with atherosclerosis [16], therefore we hypothesized that the previously found circulating miRNAs in heart failure might be related to atherosclerosis and underlying vascular disease processes.

To investigate this, we measured the previously established heart failure-related miRNA panel in another cohort of 114 heart failure patients, consisting of patients with and without atherosclerotic disease. We aimed to identify differences in circulating heart failure-related miRNA levels in heart failure patients with and without different clinical manifestations of atherosclerosis, including coronary artery disease (CAD), a medical history of stroke or transient ischemic attack (TIA) and peripheral arterial disease (PAD). In addition, we studied associations between miRNA levels and biomarkers related to atherosclerotic disease processes such as inflammation, angiogenesis and endothelial dysfunction, and we assessed the relation with the risk of rehospitalization due to cardiovascular (CV)-related causes.

## Materials and methods

### Study population

From the 1023 patients of the Coordinating Study Evaluating Outcomes of Advising and Counseling in Heart Failure (COACH), a subset of 114 randomly selected patients was studied based on the availability of plasma samples and complete biomarker measurements at baseline. The main results of the COACH study were previously published [17]. Briefly, the COACH study investigated the effect of additional specialized nurse-led support with different intensities on outcome parameters in patients with heart failure. All patients had been admitted to the hospital with symptoms of heart failure, New York Heart Association (NYHA) functional classification II to IV. Blood samples were collected shortly before discharge. Data on disease history was collected from the medical charts. Patients were divided according to the presence of 0, 1, 2 or 3 manifestations of atherosclerotic disease. The three manifestations of atherosclerotic disease consisted of CAD (defined as either a medical history of a myocardial infarction and/or a revascularization procedure by means of percutaneous coronary intervention (PCI) or coronary artery bypass grafting (CABG) surgery), TIA or stroke (defined as a medical history of either a TIA and/or a stroke) and PAD (defined as a medical history of PAD). Healthy control subjects (*n* = 10) were derived from the Telosophy study [18]. Main exclusion criteria of the control subjects were presence of heart failure, a family history of premature CV disease and known atherosclerotic disease.

### MicroRNA measurements

RNA was isolated from plasma samples using the miRCURY RNA isolation kit for bodyfluids from Exiqon (Vedbaek, Denmark). The Universal cDNA Synthesis kit (Exiqon) was used for the reversed transcription reactions. The levels of the following previous identified circulating miRNAs [7] in heart failure patients were determined in plasma from 114 heart failure patients using a customized Exiqon miRNA PCR panel; let-7i-5p, miR-16-5p, miR-18a-5p, miR-26b-5p, miR-27a-3p, miR-30e-5p, miR-106a-5p, miR-199a-3p, miR-223-3p, miR-423-5p and miR-652-3p. Circulating levels of these miRNAs were also measured in plasma samples from ten healthy control subjects, as previously described [7]. Polymerase chain reactions were performed on the LightCycler^®^ 480 (Roche Applied Science, Rotkreuz, Switzerland) with cycle settings as recommended by Exiqon. Synthetic RNA templates were used to control for isolation yield (UniSp4), cDNA synthesis (UniSp6) and PCR efficiency (UniSp3). Only miRNAs with Ct values less than 37 were included in the further analyses. The miRNA let-7a-5p was selected as best performing reference gene in the investigated cohorts, as determined by GeNorm and NormFinder (GenEx Professional software, MultiD Analyses, Sweden). Expression levels of the measured miRNAs were normalized against miRNA let-7a-5p using the GenEx Professional software and the delta Ct method was performed to obtain the relative miRNA expression levels (the Ct value of the reference miRNA was subtracted from the Ct value of the target miRNA). High miRNA expression is reflected by low delta Ct values (representing a low number of fractional cycles needed to reach the threshold of the amplified target miRNA), and low miRNA expression by high delta Ct values.

### Biomarker measurements

Plasma concentrations of the majority of atherosclerosis-related biomarkers were measured by Alere™, San Diego, CA. Competitive ELISAs on a Luminex^®^ platform were used to measure the biomarkers angiogenin, C-reactive protein (CRP), D-dimer, endothelial cell-selective adhesion molecule (ESAM), growth differentiation factor 15 (GDF-15), lymphotoxin beta receptor (LTBR), myeloperoxidase (MPO), neutrophil gelatinase-associated lipocalin (NGAL), neuropilin-1, osteopontin, pentraxin-3, polymeric immunoglobulin receptor (PIGR), receptor for advanced glycation endproducts (RAGE), syndecan-1, tumor necrosis factor alpha receptor 1 (TNFR-1), tumor necrosis factor receptor superfamily member (troy) and vascular endothelial growth receptor 1 (VEGFR-1). Endothelin-1 and interleukin-6 (IL-6) were measured by means of the high sensitive single molecule counting (SMC™) technology (RUO, Erenna^®^ Immunoassay System) by Singulex Inc. (Alameda, CA, USA). Galectin-3 was measured using the BG Medicine galectin-3 assay (BG Medicine, Waltham, MA), more extensively described elsewhere [19]. The inter- and intra-assay coefficients of variation for each of the biomarkers were previously published [20].

### Target prediction and network analysis

Potential targets of the set of circulating miRNAs were predicted using miRTarBase 6.0 [21]. Only experimentally validated targets (by means of reporter assay, western blot, microarray or next-generation sequencing) were selected to increase the reliability of the identified targets. Next, an interaction network of the overlapping miRNA targets (i.e. genes targeted by more than one of the investigated miRNAs) was created using STRING v.10 [22].

### Statistical analyses

GenEx Professional software (MultiD Analyses, Sweden) was used for the raw miRNA expression data. Other statistical analyses were conducted with R: A Language and Environment for Statistical Computing, version 3.2.0 (R Foundation for Statistical Computing, Vienna, Austria). Normally distributed variables were depicted as mean ± standard deviation and non-normally distributed variables were presented as median with the interquartile range. Differences between groups were determined using t tests for normally distributed continuous variables and Mann–Whitney *U* tests for non-normally distributed continuous variables. For binomially and categorical variables, the Chi-square test was used. Linear trend tests were used for miRNA and biomarker levels across groups and quartiles. To examine the predictive value of miRNAs for various endpoints uni- and multivariable Cox proportional hazards regression analyses were performed. *P* values of <0.05 were considered significant.

## Results

### Baseline characteristics of the study population

Baseline characteristics of the 114 hospitalized heart failure patients at time of discharge are presented in Supplementary Table 1. Patient characteristics were similar to the complete COACH population, mostly male, with a mean age of 71.1 (±10.4) years and median NT-proBNP of 3566 [1661–7848] pg/mL. Forty-four patients (39%) had no atherosclerotic disease and 70 (61%) had at least one manifestation of atherosclerotic disease. From the 114 patients, 54% showed evidence of CAD, 13% had a medical history of a previous stroke and/or TIA and 21% had PAD.

### Circulating microRNA levels in patients with heart failure compared to controls

To confirm our previous findings of lower miRNA levels in heart failure patients compared to control subjects, we compared the circulating miRNA levels of the 114 patients to a control cohort consisting of ten healthy subjects. Baseline characteristics of the control population are depicted in Supplementary Table 1. In concordance with our previous study [7], we found lower levels of the majority of the heart failure-related circulating miRNAs in heart failure patients compared to controls, with the exception of miR-423-5p and miR-16-5p showing higher and unchanged levels, respectively (Supplementary Fig. 1). Statistically significant lower levels in heart failure patients compared to healthy individuals were found for miR-18a-5p, miR-26b-5p, miR-27a-3p, miR-30e-5p, miR-199a-3p and miR-223-3p (Table 1).

**Table 1:** Circulating microRNA levels in heart failure patients (HF) compared to control subjects

Variable	Controls	HF	*P* value
*N*	10	114	
let-7i-5p	0.5 ± 0.5	0.8 ± 1	0.095
miR-16-5p	−6.1 ± 1.1	−6 ± 1.3	0.819
miR-18a-5p	1.1 ± 0.6	2.5 ± 1.1	<0.001
miR-26b-5p	2.1 ± 0.6	3.7 ± 0.9	<0.001
miR-27a-3p	−1.8 ± 0.6	−0.5 ± 1.1	<0.001
miR-30e-5p	−0.6 ± 0.6	0 ± 1.2	0.028
miR-106a-5p	−1.7 [−1.9 to 1.3]	−0.6 ± 1	0.079
miR-199a-3p	−0.6 ± 0.6	0.6 ± 1	<0.001
miR-223-3p	−5.2 [−5.5 to 4.8]	−4.5 ± 1.2	0.002
miR-423-5p	0.5 ± 1	−0.3 ± 1	0.028
miR-652-3p	0.8 ± 0.5	1.3 ± 1	0.060

Values represent the normalized (delta Ct) miRNA levels presented as mean ± standard deviation or median with interquartile range (in square brackets)

### Associations of microRNA levels with the number of different manifestations of atherosclerosis

Next, we assessed the relation between the extensiveness of atherosclerotic disease in heart failure patients and circulating miRNA levels. Patients were divided based on the number of different manifestations of atherosclerosis, including the presence of CAD, PAD and a history of stroke/TIA. For the majority of the miRNAs, the same pattern could be observed in which miRNA levels decreased in parallel with an increase of different manifestations of atherosclerotic disease (Table 2). The gradual decline in plasma levels of miR-18a-5p, miR-27a-3p, miR-199a-3p, miR-223-3p and miR-652-3p were statistically significant.

**Table 2:** Circulating microRNA levels in patients with 0, 1, 2 or 3 different manifestations of atherosclerotic disease

Variable	0	1	2	3	P-for-trend
		CAD *n* = 37	CAD *n* = 21	CAD *n* = 4	
		PAD *n* = 3	PAD *n* = 17	PAD *n* = 4	
		Stroke/TIA *n* = 3	Stroke/TIA *n* = 8	Stroke/TIA *n* = 4	
*N*	44	43	23	4	
let-7i-5p	0.9 ± 0.8	0.7 ± 1	0.8 ± 1.1	0.6 ± 1.1	0.549
miR-16-5p	−6 ± 1.1	−6.1 ± 1.4	−6 ± 1.4	−5.9 ± 1.7	0.862
miR-18a-5p	2.3 ± 0.9	2.5 ± 1.3	2.8 ± 1.1	3.6 ± 1.5	0.020
miR-26b-5p	3.8 ± 0.9	3.6 ± 0.9	3.8 ± 1.1	3.9 ± 0.6	0.718
miR-27a-3p	−0.7 ± 0.9	−0.5 ± 1.3	−0.5 ± 0.9	0.7 ± 1.7	0.014
miR-30e-5p	0 ± 1.2	−0.2 ± 1.2	0.2 ± 1.2	0.7 ± 1.8	0.178
miR-106a-5p	−0.8 ± 0.9	−0.6 ± 1.1	−0.3 ± 1.1	0.1 ± 1.4	0.078
miR-199a-3p	0.5 ± 0.9	0.5 ± 1	0.7 ± 0.9	1.5 ± 1.3	0.038
miR-223-3p	−4.8 ± 0.9	−4.4 ± 1.2	−4.1 ± 1.3	−3.5 ± 2.4	0.028
miR-423-5p	−0.1 ± 0.7	−0.4 ± 1.2	−0.5 ± 0.9	−0.3 ± 1	0.730
miR-652-3p	1.2 ± 0.8	1.2 ± 1.1	1.6 ± 0.8	2.7 ± 1.5	0.002

Number of manifestations of atherosclerotic disease are presented, including coronary artery disease (CAD), peripheral arterial disease (PAD) and stroke/transient ischemic attack (TIA). Values represent the normalized (delta Ct) miRNA levels presented as mean ± standard deviation

### Differences in circulating microRNA levels in heart failure patients with coronary artery disease, a medical history of stroke or transient ischemic attack, and peripheral arterial disease

To examine the effects of different manifestations of atherosclerotic disease on miRNA levels in more detail, we determined the differences in miRNA levels between the heart failure patients with or without CAD, a medical history of TIA/stroke and PAD. Clinical characteristics of patients belonging to these different categories of atherosclerosis are depicted in Table 3. There were no consistent trends in plasma levels of the selected miRNAs in patients with and without CAD (Supplementary Table 2A) and patients with a previous stroke or TIA (Supplementary Table 2B). In heart failure patients with PAD, several miRNA differences were found compared to heart failure patients without PAD. Plasma concentrations of all miRNAs (except for miR-423-5p) were lower in heart failure patients with PAD compared to heart failure patients without PAD (Table 4) and miR-18a-5p, miR-27a-3p, miR-30e-5p, miR-106a-5p, miR-199a-3p, miR-223-3p and miR-652-3p showed significantly lower circulating miRNA levels. Notably, heart failure patients with PAD had lower diastolic blood pressure and more patients had CAD (Table 3). Further, patients with PAD more often developed renal impairment with higher creatinine and potassium levels and a lower estimated glomerular filtration rate. The majority of the associations between the differentially expressed miRNAs and the presence of PAD remained after adjustment for these variables.

**Table 3:** Baseline characteristics of heart failure patients with and without coronary artery disease (CAD), stroke/transient ischemic attack (TIA) and peripheral arterial disease (PAD)

Variable	No CAD	CAD	*P* value	No stroke/TIA	Stroke/TIA	*P* value	No PAD	PAD	*P* value
*N*	52	62		99	15		90	24	
Demographics									
Sex (% female)	51.9 (27)	19.4 (12)	<0.001	36.4 (36)	20 (3)	0.341	37.8 (34)	20.8 (5)	0.189
Age (years)	68.8 ± 11.9	73.1 ± 8.5	0.030	71.4 ± 10.4	69.1 ± 10.2	0.432	70.6 ± 11	72.9 ± 7.2	0.240
BMI (kg/m^2^)	28.5 ± 7.1	25.6 ± 4.1	0.010	27 ± 6.1	26.6 ± 3.8	0.776	27.3 ± 6.2	25.5 ± 3.8	0.081
LVEF (%)	31.9 ± 15.2	29.9 ± 11.9	0.441	30.8 ± 13.5	30.6 ± 13.9	0.959	31.2 ± 13.6	29.3 ± 13.1	0.553
Systolic blood pressure (mmHg)	123.3 ± 23.7	114.4 ± 20.3	0.039	118.2 ± 22.6	119.7 ± 19.8	0.782	119.3 ± 22.7	114.9 ± 20.6	0.369
Diastolic blood pressure (mmHg)	70.6 ± 13	66.1 ± 13.2	0.074	68.1 ± 13.4	68.5 ± 12.6	0.898	69.6 ± 13.3	62.9 ± 11.9	0.022
Heart rate (beats/min)	75.1 ± 14.5	69.5 ± 11.2	0.028	71.5 ± 12	75.1 ± 19	0.495	72.8 ± 12.6	69.1 ± 14.5	0.258
Clinical profile, % (*n*)									
Atrial fibrillation on presentation	32.7 (17)	33.9 (21)	1	33.3 (33)	33.3 (5)	1	32.2 (29)	37.5 (9)	0.807
Orthopnea	64.7 (33)	68.9 (42)	0.793	64.9 (63)	80 (12)	0.391	65.9 (58)	70.8 (17)	0.834
Rales	92.9 (39)	88.2 (45)	0.691	90.4 (75)	90 (9)	1	93.2 (68)	80 (16)	0.182
Edema	68.6 (35)	75.8 (47)	0.523	73.5 (72)	66.7 (10)	0.811	71.9 (64)	75 (18)	0.965
Medical history, % (*n*)									
Hypertension	42.3 (22)	46.8 (29)	0.773	42.4 (42)	60 (9)	0.319	41.1 (37)	58.3 (14)	0.202
Diabetes mellitus	25 (13)	35.5 (22)	0.315	26.3 (26)	60 (9)	0.019	31.1 (28)	29.2 (7)	1
Myocardial infarction	0 (0)	88.7 (55)	<0.001	47.5 (47)	53.3 (8)	0.884	41.1 (37)	75 (18)	0.006
PCI	0 (0)	17.7 (11)	0.004	10.1 (10)	6.7 (1)	1	8.9 (8)	12.5 (3)	0.886
CABG	0 (0)	43.5 (27)	< 0.001	23.2 (23)	26.7 (4)	1	21.1 (19)	33.3 (8)	0.326
Coronary artery disease	0 (0)	100 (62)	< 0.001	52.5 (52)	66.7 (10)	0.455	47.8 (43)	79.2 (19)	0.012
Peripheral arterial disease	9.6 (5)	30.6 (19)	0.012	18.2 (18)	40 (6)	0.111	0 (0)	100 (24)	<0.001
Stroke or TIA	9.6 (5)	16.1 (10)	0.455	0 (0)	100 (15)	< 0.001	10 (9)	25 (6)	0.111
Atrial fibrillation	38.5 (20)	56.5 (35)	0.084	48.5 (48)	46.7 (7)	1	43.3 (39)	66.7 (16)	0.071
NYHA class			0.233			0.620			0.263
II	36.5 (19)	22.6 (14)		30.3 (30)	20 (3)		25.6 (23)	41.7 (10)	
III	53.8 (28)	67.7 (42)		59.6 (59)	73.3 (11)		63.3 (57)	54.2 (13)	
IV	7.7 (4)	9.7 (6)		9.1 (9)	6.7 (1)		10 (9)	4.2 (1)	
COPD	36.5 (19)	37.1 (23)	1	38.4 (38)	26.7 (4)	0.556	38.9 (35)	29.2 (7)	0.523
Medication use, % (*n*)									
ACE inhibitors or ARB	82.7 (43)	77.4 (48)	0.642	79.8 (79)	80 (12)	1	81.1 (73)	75 (18)	0.706
β-blockers	69.2 (36)	80.6 (50)	0.233	76.8 (76)	66.7 (10)	0.600	74.4 (67)	79.2 (19)	0.833
Calcium antagonists	13.5 (7)	8.1 (5)	0.529	11.1 (11)	6.7 (1)	0.943	11.1 (10)	8.3 (2)	0.984
Nitrates	25 (13)	46.8 (29)	0.027	35.4 (35)	46.7 (7)	0.576	32.2 (29)	54.2 (13)	0.081
Lipid lowering drugs	25 (13)	58.1 (36)	< 0.001	36.4 (36)	86.7 (13)	<0.001	40 (36)	54.2 (13)	0.311
Antiplatelet therapy	28.8 (15)	40.3 (25)	0.279	34.3 (34)	40 (6)	0.891	36.7 (33)	29.2 (7)	0.657
Laboratory values									
Creatinine (umol/L)	105 [83.8–132]	143 [106–181]	<0.001	116 [91.8–157]	139 [112.5–173]	0.169	114 [88–155]	142.5 [113.5–185.8]	0.012
eGFR (mL/min/1.73 m^2^)	57.9 ± 19.6	46.8 ± 18.9	0.003	52.8 ± 20	46.4 ± 19	0.244	54.1 ± 20.1	44 ± 17.4	0.020
Urea (mmol/L)	9.7 [7.7–13.9]	13.5 [9.7–19.9]	0.003	11.4 [8.4–18.4]	12.7 [9.1–15.4]	0.919	11.1 [8.2–18.8]	12.6 [9.5–15.9]	0.292
Sodium (mmol/L)	137.9 ± 4	138.3 ± 3.7	0.642	137.9 ± 3.9	139.1 ± 2.9	0.173	138 ± 3.8	138.5 ± 3.9	0.580
Potassium (mmol/L)	4.2 ± 0.6	4.4 ± 0.5	0.029	4.3 ± 0.6	4.2 ± 0.5	0.472	4.2 ± 0.5	4.5 ± 0.6	0.033
Hemoglobin (mmol/L)	8 ± 1.4	7.9 ± 1.2	0.739	7.9 ± 1.3	8.1 ± 1.6	0.889	7.8 ± 1.2	8.3 ± 1.6	0.319
BNP (pg/mL)	381 [188–1140]	514.5 [306–977]	0.194	469 [222–955]	538 [381–1530]	0.118	482 [230–984]	596.5 [230.2–1230]	0.677
NT-proBNP (pg/mL)	2825.1 [1496.4–4766.4]	4231.6 [2227.6–9781.1]	0.035	3314.4 [1611.2–7162.5]	4349.4 [2433.3–13962.5]	0.070	3360.2 [1620.7–7428]	4213 [2245.1–9949.4]	0.392

Values are presented as percentages, mean ± standard deviation or median with interquartile range (in square brackets). *BMI* body mass index, *LVEF* left ventricular ejection fraction, *PCI* percutaneous coronary intervention, *CABG* coronary artery bypass grafting, *COPD* chronic obstructive pulmonary disease, *NYHA* New York Heart Association, *ACE* angiotensin-converting enzyme, *ARB* angiotensin receptor blocker, *eGFR* estimated glomerular filtration rate, *BNP* B-type natriuretic peptide, *NT-proBNP* N-terminal pro B-type natriuretic peptide

**Table 4:** Circulating microRNA levels in heart failure patients with and without peripheral arterial disease (PAD)

Variable	No PAD	PAD	*P* value
*N*	90	24	
let-7i-5p	0.7 ± 0.9	1.1 ± 1	0.112
miR-16-5p	−6.1 ± 1.2	−5.8 ± 1.4	0.248
miR-18a-5p	2.4 ± 1.1	3.1 ± 1	0.006
miR-26b-5p	3.7 ± 0.9	4 ± 0.9	0.118
miR-27a-3p	−0.7 ± 1.1	−0.1 ± 1	0.018
miR-30e-5p	−0.2 ± 1.2	0.7 ± 1.2	0.004
miR-106a-5p	−0.7 ± 1	−0.1 ± 1	0.013
miR-199a-3p	0.4 ± 1	1 ± 0.9	0.010
miR-223-3p	−4.6 ± 1.2	−3.8 ± 1.3	0.005
miR-423-5p	−0.3 ± 1	−0.3 ± 0.9	0.986
miR-652-3p	1.1 ± 1	1.9 ± 0.8	<0.001

Values represent the normalized (delta Ct) miRNA levels presented as mean ± standard deviation

### Associations between circulating microRNAs and biomarkers

We performed target prediction analysis to identify the experimentally validated potential targets of the panel of heart failure-related miRNAs. We selected the overlapping targets (i.e. mRNAs targeted by more than one of the investigated miRNAs) and show that the majority of these targets interact which each other, as presented in the network figure (Supplementary Fig. 2). Genes with a central position in the network and multiple interactions with other target genes include FOXO1, MAPK14, CDK2, PTEN and SP1.

**Fig. 1:**
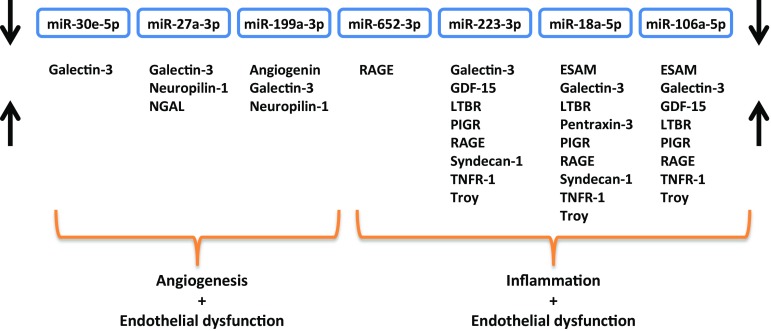
Overview of the biomarker profile corresponding to low circulating microRNA levels. The depicted miRNAs are all lower expressed in plasma of heart failure patients with PAD and multiple manifestations of atherosclerotic disease. Low levels of these miRNAs are associated with high plasma levels of several biomarkers which are related to processes involved in atherosclerosis. *ESAM* endothelial cell-selective adhesion molecule, *GDF-15* growth differentiation factor 15, *LTBR* lymphotoxin beta receptor, *NGAL* neutrophil gelatinase-associated lipocalin, *PIGR* polymeric immunoglobulin receptor, *RAGE* receptor for advanced glycation end products, *TNFR-1* tumor necrosis factor alpha receptor 1 and troy; tumor necrosis factor receptor superfamily member

Biomarkers were selected based on known associations with atherosclerosis, inflammation, angiogenesis and endothelial dysfunction. We found a total of 201 interactions between the set of biomarkers and all predicted miRNA targets (in total 213), resulting from the network analysis (Supplementary Table 3). MiRNAs differentially expressed in heart failure patients with an increasing number of manifestations of atherosclerosis and PAD were divided in quartiles based on their expression levels after which the trend with biomarker levels was determined (Table 5). A significant P-for-trend was observed for multiple biomarkers showing consistent trends of high levels in the patients with the lowest miRNA levels, including ESAM, LTBR, PIGR, pentraxin-3, troy, syndecan-1, galectin-3, NGAL, GDF-15, RAGE, TNFR-1, neuropilin-1 and angiogenin. Low levels of miR-18a-5p, miR-106a-5p and miR-223-3p were significantly associated with high levels of a variety of mainly inflammatory and endothelium-related biomarkers (ESAM, LTBR, PIGR, syndecan-1, GDF-15, RAGE, TNFR-1, pentraxin-3, galectin-3, troy), whereas low levels of miR-27a-3p and miR-199a-3p were related to high levels of biomarkers important in angiogenesis-related processes (galectin-3, neuropilin-1 and angiogenin), as summarized in Fig. 1.

**Table 5:** Circulating microRNAs significantly associated with atherosclerosis-related biomarkers

miRNA quartiles	Q1	Q2	Q3	Q4	P-for-trend
*N*	29	28	28	29	
miR-18a-5p					
ESAM	56.5 ± 16.8	60.9 ± 14.6	64 ± 21.6	65.4 ± 16.7	0.048
Galectin-3	20.4 ± 9.5	22.5 ± 8	25.3 ± 11	27.1 ± 10.4	0.007
LTBR	0.7 [0.5–1]	0.8 [0.5–1.1]	0.8 [0.5–1.4]	0.9 [0.7–1.5]	0.017
Pentraxin-3	3.8 ± 2.2	4.1 ± 1.6	4.8 ± 2.6	4.8 ± 2.3	0.045
PIGR	695.3 [413.4–1081.1]	617.4 [512.4–998.1]	774.5 [439.7–1175]	831.6 [612.6–1194.2]	0.043
RAGE	3 [2.1–5]	2.7 [1.8–4.3]	3.8 [2.7–6.1]	5 [3.7–6.3]	0.034
Syndecan-1	19.7 [13.5–25.4]	20.8 [16.2–25.5]	24.9 [16.3–32.2]	22.4 [18.8–28.2]	0.030
TNFR-1	3.1 [2.1–4.2]	3.4 [2.3–4.7]	3.3 [2.3–7.2]	4.6 [3–6.8]	0.037
Troy	0.9 [0.8–1.5]	1 [0.9–1.6]	1.2 [0.7-2]	1.5 [0.9–2.2]	0.005
miR-30e-5p					
Galectin-3	21.5 ± 7.4	22.6 ± 9.7	25.2 ± 11.8	26.3 ± 10.4	0.043
miR-27a-3p					
Galectin-3	19.9 ± 7.5	23.5 ± 7.4	23.2 ± 10.4	29.1 ± 12	0.001
Neuropilin-1	871.2 ± 234.6	953.1 ± 265.8	1007.6 ± 324.1	1091.5 ± 333.7	0.004
NGAL	107.3 [84.3–161.3]	135.4 [101.1–169.9]	151.1 [103.4–178.2]	147.7 [112.8–229.3]	0.006
miR-106a-5p					
ESAM	55.9 ± 17.1	60.7 ± 17.1	57.4 ± 14.9	72.7 ± 16.9	0.001
Galectin-3	20.6 ± 9.1	20.1 ± 5.8	24.9 ± 10.9	30.1 ± 10.3	<0.001
GDF-15	2.7 [1.8–4.4]	3 [1.9–5.3]	3.3 [1.8–6.1]	4.1 [3.2–6.4]	0.012
LTBR	0.6 [0.4–0.8]	0.8 [0.6–1.2]	0.8 [0.5–1.2]	1.2 [0.7–1.7]	<0.001
PIGR	559 [415.9–1029.5]	666.4 [421.4–1061.6]	689.6 [424.5–894.1]	1024.9 [778.2–1625.8]	<0.001
RAGE	3.1 [2.3–4.4]	3.4 [2.2–6]	3.4 [2.4–5.5]	5 [2.9–8.3]	0.011
TNFR-1	2.6 [2.1–4.1]	3.1 [2.3–4.2]	3 [2.4–5.2]	5.1 [4.1–8.6]	<0.001
Troy	0.9 [0.8–1.6]	1 [0.8–1.4]	1.2 [0.6–1.7]	1.9 [1.2–2.6]	<0.001
miR-199a-3p					
Angiogenin	3723.4 [2317.9–4907.5]	3819.8 [3252.4–5453.6]	4312.1 [2997.5–6143.4]	4595.5 [3104.5–6748.6]	0.024
Galectin-3	21.7 ± 8.4	21.8 ± 8.8	24.8 ± 11.1	27.3 ± 10.7	0.018
Neuropilin-1	871.8 ± 221.1	987 ± 330.6	1002.9 ± 260.9	1062.8 ± 350.3	0.018
miR-223-3p					
Galectin-3	19.8 ± 8.9	23.4 ± 9.3	24 ± 10.1	28.4 ± 10	0.001
GDF-15	2.8 [1.8–4]	3.1 [1.9–5.4]	3.4 [1.8–6.1]	3.7 [2.6–6.1]	0.016
LTBR	0.7 [0.5–1.1]	0.7 [0.5–1.3]	0.8 [0.5–1.3]	1 [0.7–1.6]	0.002
PIGR	593.3 [440–1040.3]	738.8 [420.9–1092.3]	695.6 [408–904.3]	976.3 [659.6–1367.6]	0.004
RAGE	3.1 [2.1–4.2]	3.6 [2.1–5.8]	3 [2.2–5]	5.7 [3.9–7.7]	0.006
Syndecan-1	19 [16.6–24.7]	23.5 [15.1–28.3]	20.9 [16.2–30]	22.8 [20.3–34.2]	0.023
TNFR-1	2.7 [2.1–3.7]	3.8 [2.3–5.3]	3.2 [2.6–5.3]	4.9 [3.2–7.5]	0.005
Troy	1 [0.8–1.6]	1.2 [0.8–1.7]	1 [0.7–1.6]	1.5 [1–2.5]	0.001
VEGFR-1	0.9 [0.1–1]	0.9 [0.6–1.2]	0.9 [0.6–1.2]	0.7 [0.5–1.7]	0.029
miR-652-3p					
RAGE	3 [2.3–4.5]	3.3 [2.1–5]	3.9 [2.1–6.2]	5 [3.1–7.5]	0.024

Biomarker values are presented in ng/mL per quartile of circulating miRNA levels, either as mean ± standard deviation or median with interquartile range (in square brackets). Quartile 1 (Q1) represents the patients with the highest miRNA levels, whereas quartile 4 (Q4) represents the patients with the lowest miRNA levels

*ESAM* endothelial cell-selective adhesion molecule, *GDF-15* growth differentiation factor 15, *LTBR* lymphotoxin beta receptor, *NGAL* neutrophil gelatinase-associated lipocalin, *PIGR* polymeric immunoglobulin receptor, *RAGE* receptor for advanced glycation endproducts, *TNFR-1* tumor necrosis factor alpha receptor 1, *troy* tumor necrosis factor receptor superfamily member, *VEGFR-1* vascular endothelial growth receptor 1

### Predictive value of circulating microRNAs and cardiovascular-related rehospitalization

**Fig. 2:**
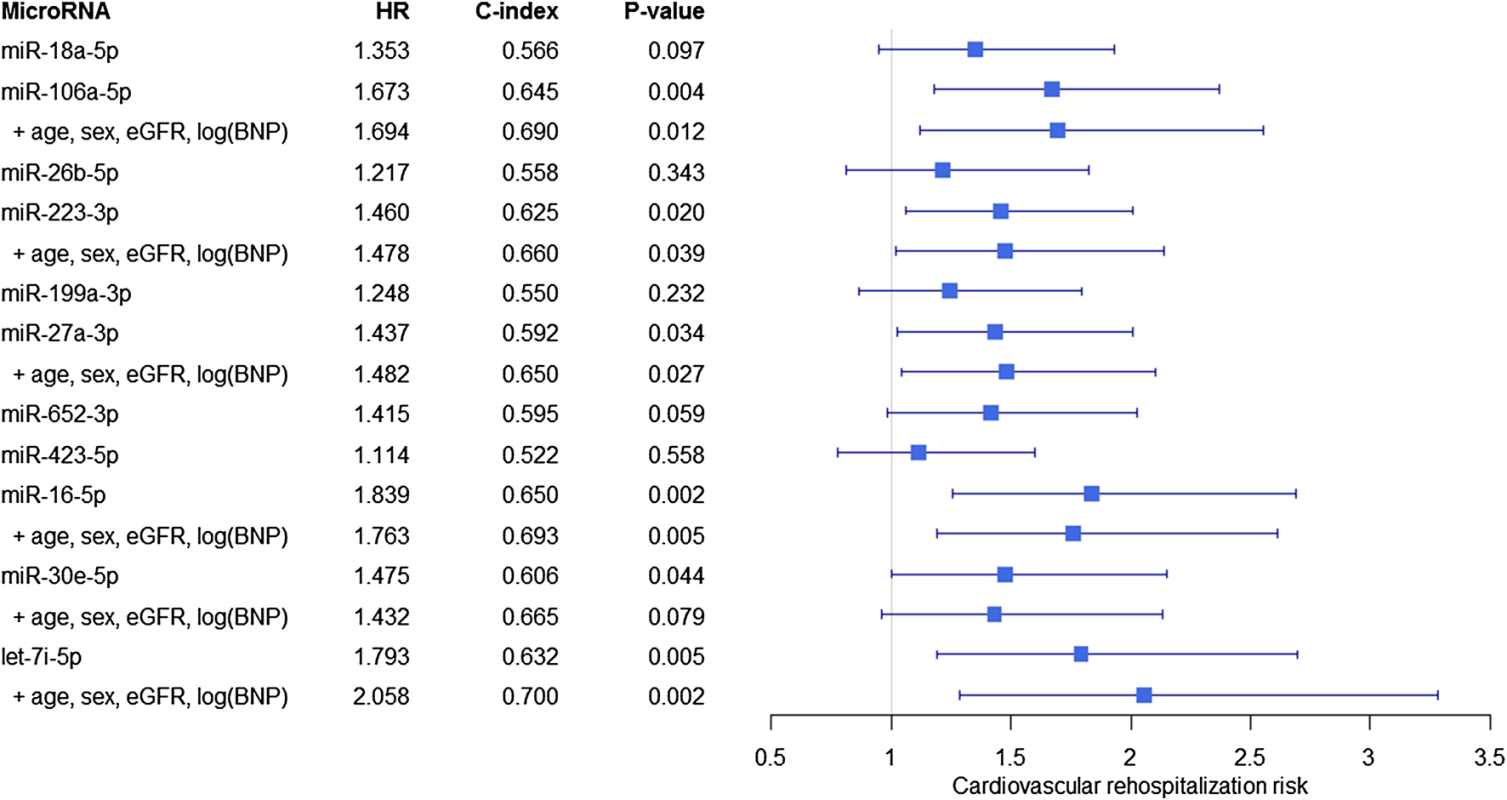
Predictive value of circulating microRNAs for cardiovascular-related rehospitalizations within 18 months. Univariable Cox proportional hazards regression analyses were performed for all circulating miRNAs. Only univariable significant miRNAs (*P* < 0.05) were added to a clinical model including age, sex, eGFR and log(BNP). This clinical model reached a C-index of 0.611 (all variables *P* > 0.05). The hazard ratio (HR) is depicted with 95% confidence interval and should be interpreted per standard deviation. C-statistics were performed to assess model performance (presented as C-index)

We studied the association between our set of established circulating miRNAs and CV-related endpoints. Within 18 months, 28 events of rehospitalization resulted from CV causes (with exclusion of heart failure), of which 18 (64%) were due to an atherosclerosis-related event (Supplementary Table 4). Univariable Cox proportional hazards analyses identified miR-106a-5p, miR-223-3p, miR-27a-3p, miR-16-5p, miR-30e-5p and let-7i-5p as significantly predictive for a CV-related rehospitalization (Fig. 2), showing consistent associations of low miRNA levels with an increased risk of reaching the endpoint. The addition of clinically relevant variables including age, sex, b-type natriuretic peptide (BNP) and estimated glomerular filtration rate (eGFR) resulted in 5 miRNAs remaining significantly predictive. C-statistics identified the model with let-7i-5p as best performing with a C-index of 0.70. The same analyses with these miRNAs and other clinical endpoints including heart failure rehospitalization and mortality did not result in similar findings. No significant associations were identified for any of the miRNAs with all-cause mortality within 18 months and only miR-106a-5p was univariable predictive for a heart failure rehospitalization and the primary combined endpoint (heart failure rehospitalization and/or death within 18 months), as presented in Supplementary Tables 5A-C. However, this association did not remain significant after adjustment for clinically relevant parameters.

## Discussion

In the present study, we confirmed our previous finding of a specific set of miRNAs that were lower expressed in patients with heart failure compared with age-matched controls. Within our group of heart failure patients, several of these heart failure-related miRNAs were lower in patients with multiple manifestations of atherosclerotic disease, and PAD in particular. These results were supported by the finding that low levels of these miRNAs were associated with high levels of several biochemical markers related to inflammation, angiogenesis and endothelial dysfunction, which are all involved in the development and progression of atherosclerosis. Finally, low levels of six of these heart failure specific miRNAs were shown to predict the risk of a CV-related readmission after a heart failure hospitalization. These findings suggest a potential involvement of these miRNAs in atherosclerosis and related disease mechanisms.

### Circulating microRNAs and peripheral arterial disease

Non-coronary atherosclerotic disease is a common comorbidity in heart failure patients and it has been shown to be an important predictor of the presence of CAD [23]. PAD in specific can be regarded to as generalized manifestation of atherosclerotic disease, which might explain why we found the most striking association between low levels of miRNAs and the presence of PAD. Few studies investigated the circulating miRNA profile in patients with PAD. Stather et al. [24] identified several downregulated circulating miRNAs related to PAD with similarities to our investigated circulating miRNA panel, including miR-16, miR-26b and miR-27b. Another study in patients with atherosclerotic abdominal aortic aneurysms found significantly upregulated miR-223 levels in atherosclerotic tissue, whereas miR-223 levels in plasma were downregulated [25], in concordance with our study.

### Associations between microRNAs and atherosclerosis-related disease mechanisms

The potential involvement of these miRNAs in atherosclerosis-related processes was further supported by the association between low levels of circulating miRNAs and elevated levels of biomarkers related to inflammation, angiogenesis and endothelial dysfunction. Interestingly, these processes are all well-described disease mechanisms in both atherosclerosis [16, 26] and heart failure [27, 28].

Especially miR-18a-5p, miR-106a-5p and miR-223-3p were associated with a high number of mainly inflammatory and endothelium-related biomarkers, including ESAM, RAGE and pentraxin-3. Various roles for these biomarkers have been described, including migration of neutrophils and macrophages [29], leukocyte adhesion [30], endothelial dysfunction and vascular homeostasis [31]. The associations between these biomarkers and several heart failure-related miRNAs coincide with previous associations of miR-18a-5p, miR-106a-5p and miR-223-3p with inflammation and endothelial-related processes. In endothelial cells, miR-18a (part of the miR-17~92 cluster) was mainly described as anti-angiogenic [32], although a recent study reported that this cluster was required for endothelial cell proliferation and angiogenic sprouting after VEGF stimulation [10]. This suggests that the miR-17~92 cluster exhibits complex roles in endothelial cell function and angiogenesis, although the precise understanding of the underlying mechanisms warrants further investigation. MiR-223 is a well-known inflammation-related miRNA and is abundant in platelets, leukocytes and endothelial-derived microvesicles [11]. Besides its anti-angiogenic properties it was shown that miR-223 can function as potential contributor to the quiescence of endothelial cells [12]. Furthermore, MiR-106a has been associated to macrophage activation, suggesting involvement in inflammation [13].

We showed that low levels of miR-27a-3p and miR-199a-3p were associated with angiogenesis-related markers including angiogenin, neuropilin-1 and galectin-3. MiR-27a is present in endothelial cells and was previously described as key regulator of endothelial cell sprouting and angiogenesis [14], suggesting a substantial involvement in vascular dysfunction. MiR-199a-3p is mainly described as hypoxia-related miRNA and can function as promoter of metastasis and angiogenesis [15]. A potential role for miR-199a-3p in angiogenesis is also reflected in the present study by the observed association with angiogenesis-related markers.

Our target prediction and network analyses also imply involvement of the investigated miRNAs in atherosclerosis-related processes, since targets as FOXO1 and CDK2 were previously shown to have key roles in the development of atherosclerosis, including angiogenesis, oxidative stress and proliferation of smooth muscle cells [33, 34]. Interestingly, both FOXO1 and MAPK14—another important node in our network—were also implicated in the development of heart failure [35, 36], therefore the identified targets may reflect key regulating mechanisms in both atherosclerotic disease and heart failure.

### Relation of circulating microRNAs to rehospitalization due to cardiovascular causes

Besides the associations with the clinical phenotype and biochemical profile of atherosclerosis, we found very consistent associations between low levels of several miRNAs and CV-related rehospitalizations within 18 months, while no relations with other clinical endpoints including heart failure rehospitalization and mortality were found. Interestingly, most of the CV readmissions were related to atherosclerosis, suggesting that these miRNAs are able to predict the risk of atherosclerosis-related rehospitalizations in patients with heart failure.

Hospital readmission after a hospitalization for acute heart failure is a major problem and although biomarkers can predict response to acute heart failure treatment [37], few valuable predictors of long-term outcome besides the natriuretic peptides have been proposed so far [38]. Moreover, studies investigating the predictive value of circulating miRNAs in (acute) heart failure patients in relation to adverse outcome are scarce. Two studies identified miR-423-5p as prognostic biomarker for a hospital readmission [39] and all-cause mortality [7] in acute heart failure patients, but in the current study this miRNA did not predict a CV-related rehospitalization. Here, we identified let-7i-5p as strongest predictor of CV-related rehospitalizations and although there is no literature specifically addressing the relation of let-7i-5p with clinical outcome, the let-7 family has been described before in relation to CV disease [40]. Not all miRNAs with significant predictive value for CV rehospitalization overlap with the miRNAs found to be related to the atherosclerotic phenotype and vice versa, which may indicate that some miRNAs mainly reflect processes underlying atherosclerosis while others have a stronger association with progressing disease and outcome parameters. Nevertheless, we found a highly consistent pattern of lower miRNA levels associated with the atherosclerotic disease phenotype as well as an increased risk of CV rehospitalizations.

### Low circulating microRNA levels; increased uptake or decreased secretion?

The consistent pattern of decreased circulating miRNA levels associated with different aspects of atherosclerotic disease is intriguing and leads to questions regarding their biological role in the circulation. One possible explanation for these low miRNA levels might lie in the increased uptake by recipient cells. It has been shown that circulating miRNAs can function in cell-to-cell communication and that recipient cells can engulf vesicle encapsulated miRNAs which consequently alters important cell functions [1]. In atherosclerosis, Zernecke et al. demonstrated in vitro that miR-126-enriched apoptotic bodies produced by endothelial cells can be taken up by vascular cells to regulate VEGF [41]. On the other hand, a diminished release of miRNA-enriched vesicles could also lead to downregulated miRNA levels in plasma. Since increased angiogenesis is associated with plaque progression and instability in atherosclerosis, it has been speculated that a reduced export of angiogenic miRNAs outside cells might inhibit pro-angiogenic signaling [42]. Indeed, in serum of patients with CAD, it has been shown that extracellular vesicles are loaded with less CAD-related miRNAs in comparison to healthy subjects [43]. However, the majority of the extracellular miRNAs are vesicle-free and bound to Ago proteins, of which no evidence of miRNA trafficking is currently available. Therefore, further research is needed to unravel the precise underlying mechanisms of reduced circulating miRNA levels in patients with heart failure and atherosclerosis.

### Study limitations

The limitations of this study should be acknowledged. First, although very consistent miRNA patterns were found, the studied patient population was relatively small. Second, the associations of heart failure-related circulating miRNAs with atherosclerosis and their role in common disease mechanisms such as vascular dysfunction should be further investigated in experimental settings to determine causal links.

## Conclusions

Although the precise functions of circulating miRNAs in heart failure are still elusive, this study proposes a link between downregulated heart failure-related miRNAs and the presence of atherosclerosis and provides insight into potential related pathophysiological mechanisms including angiogenesis, inflammation and endothelial dysfunction. Further, we show the predictive value of these circulating miRNAs for the risk of a CV rehospitalization in heart failure patients. Future studies may elucidate the involvement of circulating miRNAs in heart failure and atherosclerosis-related disease pathways, potentially leading to novel biomarkers and drug targets.

## Electronic supplementary material

Below is the link to the electronic supplementary material.

Supplementary material 1 (DOCX 700 KB)
