# Supplementary material for: Low circulating microRNA levels in heart failure patients are associated with atherosclerotic disease and cardiovascular-related rehospitalizations
Source: Clin Res Cardiol. 2017 Mar 14;106(8):598–609. doi: 10.1007/s00392-017-1096-z (PMC5529487; doi:10.1007/s00392-017-1096-z)
Supplement: Supplementary file 1 — Supplementary material 1 (DOCX 700 KB) [file 392_2017_1096_MOESM1_ESM.docx]

**Supplementary Material**

**Low circulating microRNA levels in heart failure patients are associated with atherosclerotic disease and cardiovascular related rehospitalizations**
Eline L. Vegter^1^, MD, Ekaterina S. Ovchinnikova^1,2^, PhD, Dirk J. van Veldhuisen^1^, MD, PhD, Tiny Jaarsma^3^, PhD, Eugene Berezikov^2^, PhD, Peter van der Meer^1^, MD, PhD and Adriaan A. Voors^1*^, MD, PhD
 **Clinical Research in Cardiology**

^1^ Department of Cardiology, University Medical Center Groningen, Hanzeplein 1, 9713 GZ, Groningen, The Netherlands;

^2^ European Research Institute for the Biology of Ageing, University of Groningen, Antonius Deusinglaan 1, 9713 AV, Groningen, The Netherlands;

^3^ Faculty of medical and health sciences, University of Linkoping, 581 83 Linköping, Sweden

* Address for correspondence:
Adriaan A. Voors, MD, PhD,
Department of Cardiology, AB31
University Medical Center Groningen
Hanzeplein 1
9713 GZ, Groningen
The Netherlands
Tel: +31503612355
Fax: +31503614391
Email: [a.a.voors@umcg.nl](mailto:a.a.voors@umcg.nl)

**Supplementary Tables and Figures**

**Supplementary Table 1. Clinical characteristics of the total COACH cohort [1], the study population and control subjects [2]**

|  | |  |  |
| --- | --- | --- | --- |
|  |  |  |  |
| **Variable** | **Total COACH cohort** | **Study population** | **Control cohort** |
| **N =** | **1023** | **114** | **10** |
| **Demographics** |  |  |  |
| Sex (% female, n) | 38 (384) | 34 (39) | 30 (3) |
| Age (years) | 71±11 | 71±10 | 67±6 |
| BMI (kg/m2) | 27±5 | 27±6 | 25±3 |
| LVEF (%) | 34±14 | 31±14 |  |
| Systolic blood pressure (mmHg) | 118±21 | 118±22 | 136±15 |
| Diastolic blood pressure (mmHg) | 68±12 | 68±13 | 81±9 |
| Heart rate (beats/min) | 75±14 | 72±13 | 67±7 |
| **Medical History, % (n)** |  |  |  |
| Hypertension | 43 (439) | 45 (51) | 30 (3) |
| Diabetes mellitus | 28 (289) | 31 (35) | 10 (1) |
| Myocardial infarction | 43 (436) | 48 (55) | 0 (0) |
| PCI | 11 (108) | 10 (11) |  |
| CABG | 16 (165) | 24 (27) |  |
| Coronary artery disease | 48 (492) | 54 (62) |  |
| Peripheral arterial disease | 16 (168) | 21 (24) |  |
| Stroke or TIA | 16 (164) | 13 (15) | 0 (0) |
| Renal disease | 8 (78) | 9 (10) |  |
| Atrial fibrillation | 36 (372) | 48 (55) | 0 (0) |
| COPD | 27 (268) | 37 (42) |  |
| NYHA class |  |  |  |
| II | 50 (513) | 29 (33) |  |
| III | 46 (461) | 61 (70) |  |
| IV | 4 (34) | 9 (10) |  |
| **Medication Use, % (n)** |  |  |  |
| ACE inhibitors or ARB | 83 (847) | 80 (91) | 30 (3) |
| β-blockers | 66 (677) | 75 (86) | 10 (1) |
| Calcium antagonists | 16 (162) | 11 (12) | 10 (1) |
| Nitrates | 32 (324) | 37 (42) |  |
| Lipid lowering drugs | 38 (388) | 43 (49) | 10 (1) |
| **Laboratory Values** |  |  |  |
| Creatinine (umol/L) | 113 [91-144] | 119 [95-157] |  |
| Urea (mmol/L) | 10.7 [8.1-15.2] | 11.8 [8.6-18.1] |  |
| eGFR (mL/min/1.73 m^2^) | 55.2±21.1 | 51.9±19.9 |  |
| Sodium (mmol/L) | 139±4 | 138±4 |  |
| Potassium (mmol/L) | 4.2±0.5 | 4.3±0.6 |  |
| BNP (pg/mL) | 447 [195-889] | 493 [226-1035] |  |
| NT-proBNP (pg/mL) | 2520 [1289-5508] | 3566 [1661-7848] | 49 [26-67] |

Values are presented either as percentages, mean ± standard deviation or median with interquartile ranges (in square brackets). BMI indicates body mass index; LVEF, left ventricular ejection fraction; PCI, percutaneous coronary intervention; CABG, coronary artery bypass grafting; TIA, transient ischemic attack; COPD, chronic obstructive pulmonary disease; NYHA, New York Heart Association; ACE, angiotensin-converting enzyme; ARB, angiotensin receptor blocker; eGFR, estimated glomerular filtration rate; BNP, B-type natriuretic peptide and NT-proBNP, N-terminal pro B-type natriuretic peptide. **Supplementary Table 2A. Circulating microRNA levels in heart failure patients with and without coronary artery disease (CAD)**

| **Variable** | **No CAD** | **CAD** | **P-value** |
| --- | --- | --- | --- |
| N = | 52 | 62 |  |
| **let-7i-5p** | **1±0.9** | **0.6±1** | **0.036** |
| miR-16-5p | -5.9±1.2 | -6.2±1.3 | 0.321 |
| miR-18a-5p | 2.4±0.9 | 2.6±1.3 | 0.305 |
| miR-26b-5p | 3.8±0.9 | 3.7±1 | 0.391 |
| miR-27a-3p | -0.6±0.9 | -0.5±1.3 | 0.442 |
| miR-30e-5p | 0.1±1.2 | 0±1.2 | 0.629 |
| miR-106a-5p | -0.7±0.8 | -0.5±1.1 | 0.154 |
| miR-199a-3p | 0.6±0.9 | 0.5±1.1 | 0.963 |
| **miR-223-3p** | **-4.7±0.9** | **-4.2±1.4** | **0.021** |
| miR-423-5p | -0.1±0.8 | -0.5±1.1 | 0.054 |
| miR-652-3p | 1.3±0.8 | 1.3±1.1 | 0.931 |

Values represent the normalized (delta Ct) miRNA levels presented as mean ± standard deviation. **Supplementary Table 2B. Circulating microRNA levels in heart failure patients with and without a history of stroke or transient ischemic attack (TIA)**

| **Variable** | **No stroke/TIA** | **Stroke/TIA** | **P-value** |
| --- | --- | --- | --- |
| N = | 99 | 15 |  |
| let-7i-5p | 0.8±0.9 | 0.6±1.2 | 0.458 |
| miR-16-5p | -6±1.2 | -6.1±1.6 | 0.849 |
| miR-18a-5p | 2.5±1.1 | 2.9±1.4 | 0.309 |
| miR-26b-5p | 3.7±0.9 | 3.6±1.1 | 0.783 |
| miR-27a-3p | -0.6±1.1 | -0.3±1.3 | 0.425 |
| miR-30e-5p | 0±1.2 | 0±1.5 | 0.929 |
| miR-106a-5p | -0.6±1 | -0.5±1.1 | 0.797 |
| miR-199a-3p | 0.5±1 | 0.7±1.1 | 0.466 |
| miR-223-3p | -4.5±1.2 | -4.4±1.6 | 0.989 |
| miR-423-5p | -0.3±1 | -0.5±0.9 | 0.355 |
| miR-652-3p | 1.2±0.9 | 1.7±1.2 | 0.148 |

Values represent the normalized (delta Ct) miRNA levels presented as mean ± standard deviation.

**Supplementary Table 3. Interaction between atherosclerosis related biomarkers and predicted microRNA targets**

| **Biomarker** | **Number of linked targets** |
| --- | --- |
| VEGFR-1 | 20 |
| Galectin-3 | 14 |
| GDF15 | 12 |
| Syndecan-1 | 16 |
| TNFR-1 | 13 |
| CRP | 1 |
| PIGR | 2 |
| Osteopontin | 10 |
| LTBR | 1 |
| Pentraxin-3 | 1 |
| Neuropilin-1 | 9 |
| Angiogenin | 1 |
| Troy | 0 |
| NGAL | 3 |
| Rage | 3 |
| ESAM | 5 |
| MPO | 0 |
| Endothelin-1 | 31 |
| Interleukin-6* | 58 |
| D-dimer | 1 |

The number of interactions between the investigated biomarkers and the miRNA targets resulting from the network analysis are presented. Only targets with experimental validation as predicted by miRTarBase were selected. Interactions were computed by STRING with use of experimental data, text mining of scientific text and genomic features (genomic neighborhood, fusion-fission events, occurrence within the same metabolic pathways and co-expression). * The biomarker interleukin-6 was also identified as one of the predicted targets. CRP indicates C-reactive protein; ESAM, endothelial cell-selective adhesion molecule; GDF-15, growth differentiation factor 15; LTBR, lymphotoxin beta receptor; MPO, myeloperoxidase; NGAL, neutrophil gelatinase-associated lipocalin; PIGR, polymeric immunoglobulin receptor; RAGE, receptor for advanced glycation endproducts; TNFR-1, tumor necrosis factor alpha receptor 1; troy, tumor necrosis factor receptor superfamily member and VEGFR-1, vascular endothelial growth receptor 1.

**Supplementary Table 4. Causes of cardiovascular related rehospitalization (n=28)**

| **Event** |  |  | **N =** |
| --- | --- | --- | --- |
| **Atherosclerosis related** |  |  |  |
| Angina pectoris |  |  | 6 |
| CVA |  |  | 4 |
| Peripheral arterial disease | |  | 2 |
| Percutaneous transluminal angioplasty femoral artery | | | 2 |
| Carotid endarterectomy |  |  | 1 |
| Thrombosis mesenteric artery | |  | 1 |
| CABG |  |  | 1 |
| Acute coronary syndrome |  |  | 1 |
|  |  |  |  |
| **Non-atherosclerosis related** | |  |  |
| Dehydration |  |  | 3 |
| Syncope |  |  | 3 |
| ICD implantation |  |  | 2 |
| Atrial flutter |  |  | 1 |
| Atrial fibrillation |  |  | 1 |
|  |  |  |  |

**Supplementary Table 5A. Predictive value of circulating microRNAs for the primary endpoint (heart failure rehospitalization and/or death within 18 months)**

|  | |  |  |
| --- | --- | --- | --- |
| **Primary endpoint (55 events)** | |  |  |
|  | **Hazard ratio (95% CI)** | **Harrell's C-index** | **P-value** |
| let-7i-5p | 1.169 (0.884-1.546) | 0.551 | 0.273 |
| miR-16-5p | 1.237 (0.943-1.621) | 0.571 | 0.124 |
| miR-18a-5p | 1.186 (0.918-1.532) | 0.568 | 0.193 |
| miR-26b-5p | 0.881 (0.653-1.188) | 0.532 | 0.405 |
| miR-27a-3p | 1.064 (0.833-1.358) | 0.540 | 0.620 |
| miR-30e-5p | 1.219 (0.927-1.602) | 0.557 | 0.157 |
| **miR-106a-5p** | **1.378 (1.065-1.781)** | **0.609** | **0.015** |
| miR-199a-3p | 1.075 (0.832-1.390) | 0.529 | 0.581 |
| miR-223-3p | 1.245 (0.984-1.576) | 0.601 | 0.069 |
| miR-423-5p | 0.945 (0.730-1.228) | 0.540 | 0.683 |
| miR-652-3p | 1.085 (0.835-1.409) | 0.535 | 0.542 |
|  |  |  |  |
| miR-106a-5p* | 1.150 (0.871-1.518) | 0.677 | 0.325 |
| *corrected for sex, age, eGFR and log(BNP) | | | |

**Supplementary Table 5B. Predictive value of circulating microRNAs for all-cause mortality within 18 months**

| **All-cause mortality (35 events)** | |  |  |
| --- | --- | --- | --- |
|  | **Hazard ratio (95% CI)** | **Harrell's C-index** | **P-value** |
| let-7i-5p | 1.139 (0.8145-1.594) | 0.525 | 0.446 |
| miR-16-5p | 1.132 (0.820-1.562) | 0.537 | 0.450 |
| miR-18a-5p | 1.048 (0.754-1.457) | 0.518 | 0.779 |
| miR-26b-5p | 0.820 (0.576-1.167) | 0.557 | 0.271 |
| miR-27a-3p | 1.048 (0.768-1.430) | 0.544 | 0.768 |
| miR-30e-5p | 1.083 (0.7842-1.497) | 0.528 | 0.627 |
| miR-106a-5p | 1.135 (0.838-1.536) | 0.572 | 0.414 |
| miR-199a-3p | 1.050 (0.762-1.447) | 0.528 | 0.767 |
| miR-223-3p | 1.107 (0.814-1.506) | 0.565 | 0.517 |
| miR-423-5p | 0.959 (0.690-1.332) | 0.517 | 0.801 |
| miR-652-3p | 0.983 (0.713-1.355) | 0.481 | 0.914 |

**Supplementary Table 5C. Predictive value of circulating microRNAs for heart failure rehospitalization within 18 months**

| **HF rehospitalization (38 events)** | |  |  |
| --- | --- | --- | --- |
|  | **Hazard ratio (95% CI)** | **Harrell's C-index** | **P-value** |
| let-7i-5p | 1.031 (0.738-1.439) | 0.528 | 0.858 |
| miR-16-5p | 1.185 (0.854-1.643) | 0.569 | 0.310 |
| miR-18a-5p | 1.098 (0.800-1.508) | 0.561 | 0.562 |
| miR-26b-5p | 0.849 (0.590-1.221) | 0.541 | 0.377 |
| miR-27a-3p | 1.060 (0.790-1.423) | 0.532 | 0.698 |
| miR-30e-5p | 1.171 (0.841-1.630) | 0.551 | 0.350 |
| **miR-106a-5p** | **1.383 (1.017-1.882)** | **0.600** | **0.039** |
| miR-199a-3p | 1.125 (0.827-1.530) | 0.541 | 0.453 |
| miR-223-3p | 1.236 (0.930-1.643) | 0.596 | 0.144 |
| miR-423-5p | 0.958 (0.700-1.312) | 0.543 | 0.789 |
| miR-652-3p | 1.181 (0.866-1.609) | 0.552 | 0.293 |
|  |  |  |  |
| miR-106a-5p* | 1.205 (0.868-1.675) | 0.682 | 0.266 |
| *corrected for sex, age, eGFR and log(BNP) | | | |

Univariable Cox proportional hazards regression analyses were performed for all circulating miRNAs. Only univariable significant miRNAs (p<0.05) were added to a clinical model including age, sex, eGFR and log(BNP). This clinical model reached a C-index of 0.611 (all variables p>0.05). The hazard ratio (HR) is depicted with 95% confidence interval and should be interpreted per standard deviation. C-statistics were performed to assess model performance (presented as C-index).


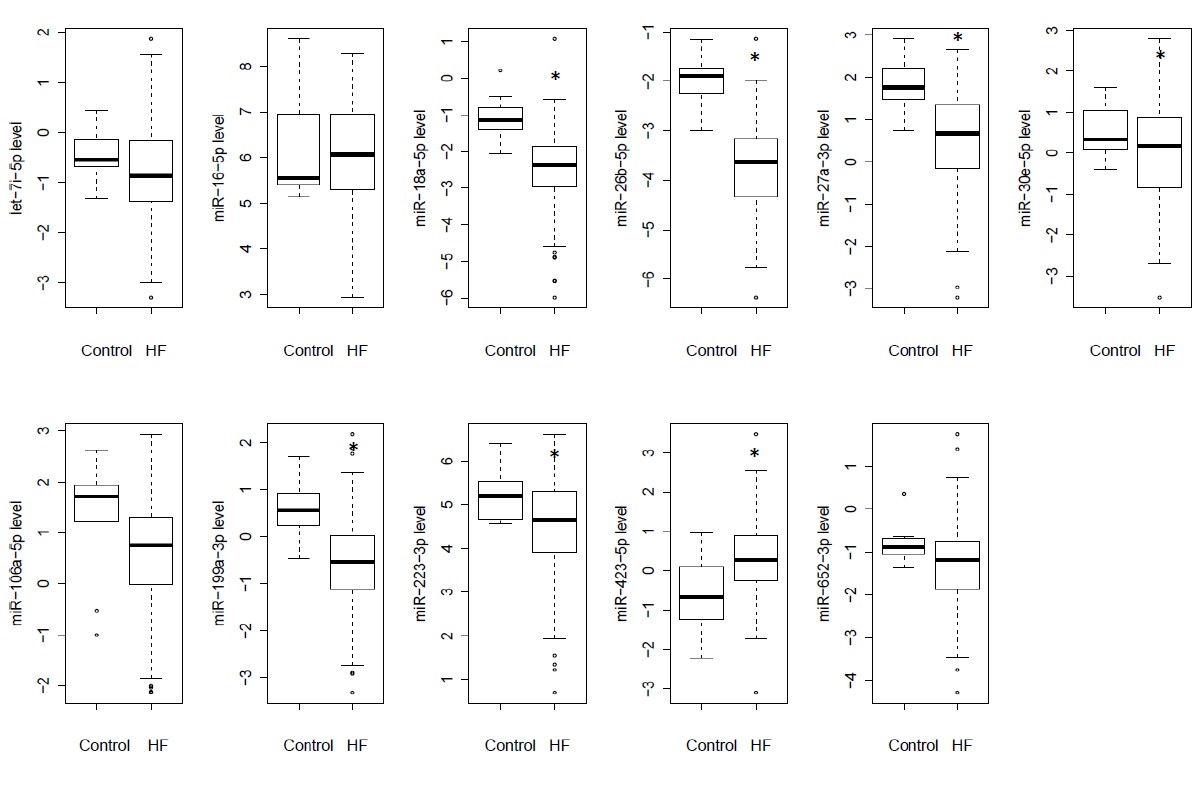
**Supplementary Figure 1. Circulating microRNA levels from heart failure patients (HF) and control subjects**

Boxplots of the normalized (delta -Ct) miRNA levels are depicted with the median, minimum, maximum, first and third quartile. * indicates significant differences between groups (p<0.05).


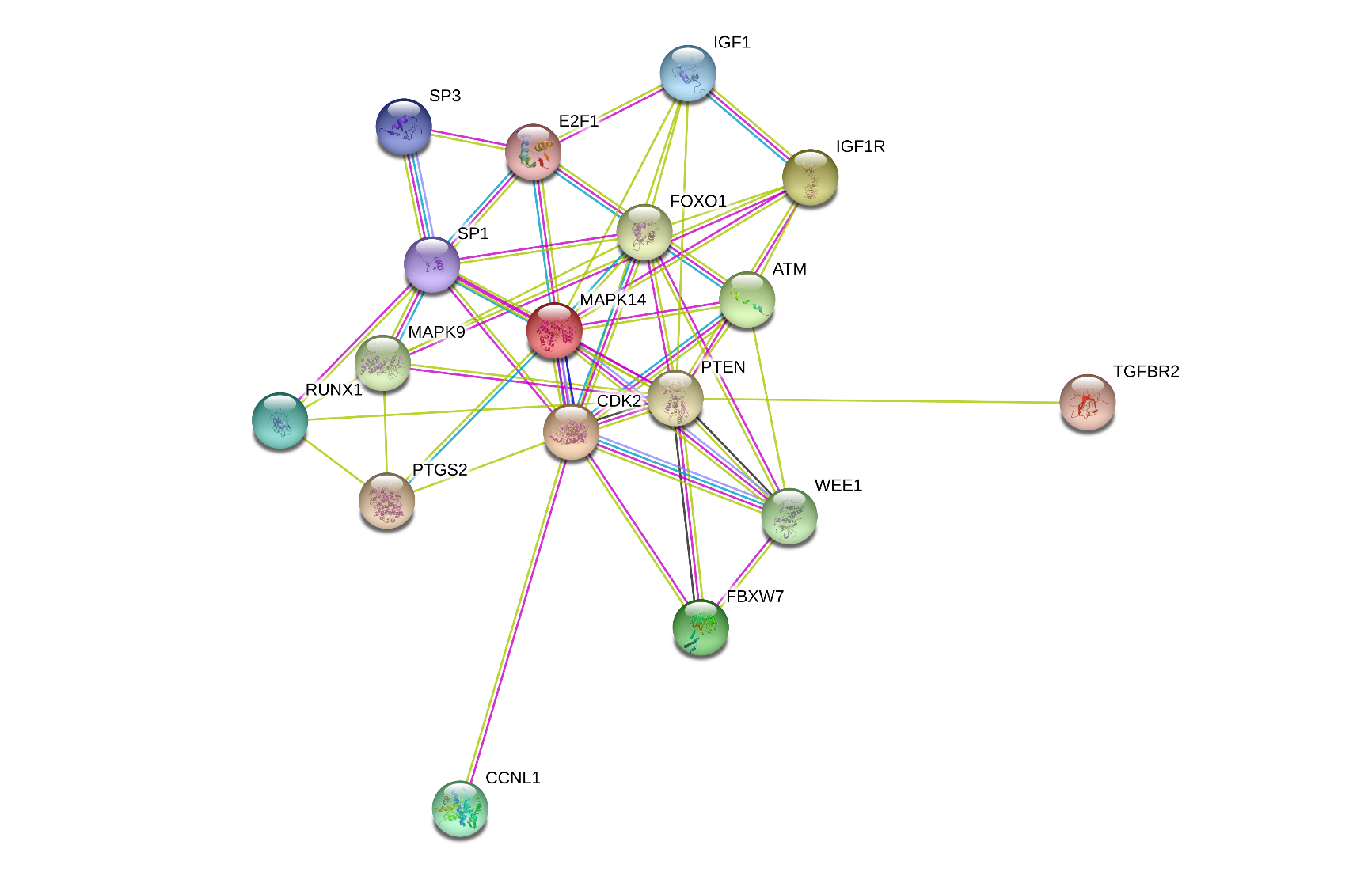
**Supplementary Figure 2.** **Network of overlapping microRNA targets**

Overlapping miRNA targets are depicted in nodes. Lines represent interactions with other target genes, as determined by STRING with use of experimental data, text mining of scientific text and genomic features (genomic neighborhood, gene fusions, occurrence within the same metabolic pathways and co-expression). A light blue line represents a known interaction from curated databases; pink, experimentally determined interaction; dark green, gene neighborhood; red, gene fusion; dark blue, gene co-occurrence and light green, text mining.

**References**

1. Jaarsma T, van der Wal MH, Lesman-Leegte I, Luttik ML, Hogenhuis J, Veeger NJ, Sanderman R, Hoes AW, van Gilst WH, Lok DJ, Dunselman PH, Tijssen JG, Hillege HL, van Veldhuisen DJ, Coordinating Study Evaluating Outcomes of Advising and Counseling in Heart Failure (COACH) Investigators (2008) Effect of moderate or intensive disease management program on outcome in patients with heart failure: Coordinating Study Evaluating Outcomes of Advising and Counseling in Heart Failure (COACH). Arch Intern Med 168:316-324

2. Wong LS, Huzen J, de Boer RA, van Gilst WH, van Veldhuisen DJ, van der Harst P (2011) Telomere length of circulating leukocyte subpopulations and buccal cells in patients with ischemic heart failure and their offspring. PLoS One 6:e23118
